# Human Susceptibility to UVB-Induced Immunosuppression Is Associated with UVB-Induced Activation of the DAMP Pathway in the Skin

**DOI:** 10.3390/cells15191732

**Published:** 2026-09-23

**Authors:** Carlos Alberto Mier-Aguilar, Tyler Downing, Mohammad Asif Sherwani, Yuko Tsuruta, Mohammad Saleem, David K. Crossman, Sejong Bae, Andrzej T. Slominski, Craig A. Elmets, Nabiha Yusuf, Hui Xu

**Affiliations:** 1 Department of Dermatology, Heersink School of Medicine, University of Alabama at Birmingham, Birmingham, AL 35294, USA; 2 Department of Genetics, University of Alabama at Birmingham, Birmingham, AL 35294, USA; 3 Department of General Medicine and Population Sciences, Heersink School of Medicine, University of Alabama at Birmingham, Birmingham, AL 35294, USA

**Keywords:** ultraviolet radiation, skin, immune suppression

## Abstract

Background: Although ultraviolet B (UVB) radiation-induced immunosuppression is considered a key risk factor for skin cancer, individual susceptibility varies, and its mechanisms remain not fully understood. Objective: The current study collected skin biopsies from healthy volunteers before and after UVB radiation and linked UVB-induced effects in the skin to immunosuppression in the same individuals. Methods: A total of 62 healthy volunteers were recruited and randomly assigned to three groups: negative, positive, and UVB-exposed. Diphenylcyclopropenone was applied topically to induce contact hypersensitivity (CHS) responses. Skin punch biopsies were collected before and after UVB for analysis. Results: UVB exposure significantly suppressed CHS compared to the positive control. However, among 26 individuals, 7 (27%) were resistant (UVB-R), while the rest were susceptible (UVB-S) to UVB-induced immunosuppression. RNA-seq analysis of skin biopsies showed that UVB induced a higher level of immunosuppressive inflammation in UVB-S than in UVB-R individuals. Further analysis showed that UVB significantly activated the damage-associated molecular patterns only in UVB-S, but not in UVB-R individuals. Conclusion: Given the role of DAMP pathways in the development of inflammation, the results reveal a previously unrecognized mechanism: UVB-induced activation of DAMP pathways is linked to UVB-induced immunosuppressive inflammation in skin and susceptibility to immunosuppression in humans.

## 1. Introduction

The American Cancer Society acknowledges that most skin cancers are a direct result of exposure to the UV rays in sunlight. The incidence of skin cancer in the United States has doubled from 1992 to 2012 [1]. In fact, organ transplant recipients who are treated with immunosuppressive medications have a greatly increased risk (up to 100 times) of UV-induced skin cancers, and the tumors that do develop behave more aggressively [2,3,4,5]. Therefore, understanding mechanisms of UV-induced immunosuppression is important for the prevention and treatment of skin cancers.

Susceptibility to UVB-induced immunosuppression varies among individuals [6,7,8]. Genetic variations are commonly considered factors for UVB susceptibility in humans [9,10,11]. Similar results have also been reported in animal studies [12,13]. It remains unclear why some people are susceptible to UVB-induced skin damage and to skin cancers. Mechanisms for human susceptibility to UVB-induced immunosuppression are understudied.

UVB induces DNA damage in the skin, which triggers and initiates inflammation and immunosuppression [14,15,16,17]. UVB-induced inflammation in the skin is complex and consists of immunosuppressive mediators such as PGE2, IL-10, and PD-L1 [18,19,20,21,22,23]. Animal studies show that enhanced repair of UVB-induced DNA damage can reduce UVB-induced immunosuppressive inflammation and consequently inhibit immunosuppression [17,24,25]. It remains to be fully defined how DNA damage induces inflammation in UVB-exposed skin. Animal studies show that damage-associated molecular patterns (DAMPs) are activated by UVB and are attributed to UVB-induced inflammation and immunosuppression [7,26,27]. To our knowledge, limited information is available on the association of UVB-induced activation of the DAMP pathway with immunosuppressive inflammation and susceptibility to UVB-induced immunosuppression in humans.

We conducted a clinical study to examine UVB-induced immunosuppression and characterize UVB-induced effects on the skin in the same individuals. The study intended to link UVB-induced effects in the skin to susceptibility to immunosuppression and explore the mechanism of action. Bioinformatic analysis of RNA sequencing data from skin biopsies shows that individual susceptibility to UV-induced immunosuppression is linked to UVB-induced activation of the DAMP pathway and immunosuppressive inflammation in the skin. Interestingly, UVB-induced DNA damage/repair and cell cycle/death pathways in the skin do not appear to be associated with UVB susceptibility. The current study has added insights into the mechanisms for human susceptibility to UVB-induced immunosuppression and warrants further investigation.

## 2. Materials and Methods

### 2.1. Participant Recruitment

Eligible study participants were healthy adults between the ages of 18 and 35 with Fitzpatrick Skin Type I-III. Key exclusion criteria included a recent history of vitiligo, melasma, or other disorders of pigmentation; a history of eczema, psoriasis, or contact dermatitis; a history of photosensitivity disorders; any type of previous skin cancer; women who were lactating, pregnant, or planning to be pregnant; participants with an organ transplant, an immunodeficiency disorder, or taking an immunosuppressive drug.

They were pre-screened to assess their Fitzpatrick Skin Type and review the inclusion/exclusion criteria. After obtaining consent and screening, eligible participants were randomized to one of three groups in a 2:2:3 ratio. A total of 72 participants were eligible and recruited for the study. Participant demographic and phenotypic data were collected using the REDCap electronic data capture platform (Vanderbilt University). REDCap was used to administer standardized questionnaires, including the Fitzpatrick Skin Type Questionnaire and additional demographic items (age, gender, race, ethnicity, and clinical characteristics). The study (IRB protocol # 300007159) was approved by the Institutional Review Board of the University of Alabama at Birmingham and was registered with ClinicalTrials.gov (NCT05020496).

### 2.2. Study Design

The study design and participant characteristics are presented in Table 1. The experimental protocol and sample size (Scheme 1) were based on previous human studies [6,10,28].

UVB exposure: Groups 1 and 2 were not exposed to UVB, while Group 3 was subjected to UVB. Six one-inch squares of skin on the left upper buttock were exposed to gradually increasing doses of UVB light to determine the minimal erythema dose (MED) [29]. The starting dose for each participant varied depending on their clinical Fitzpatrick Skin Type. Skin redness was measured using a colorimeter 24 h after UVB. We compared UVB-exposed skin to non-irradiated skin to calculate the MED [29]. Based on the MED, two marked skin sites on the right upper buttock were irradiated with 2 MED of UVB 4 times on 4 consecutive days.

Skin biopsy: Two 6 mm punch skin biopsies were collected: one from the non-UVB-exposed area as a control and the other from the UVB-irradiated area 24 h after UVB. Each specimen was divided in half, with one half placed in formalin at room temperature and the other placed in RNAlater^TM^ Stabilization Solution (Invitrogen, Carlsbad, CA, USA) at −20 °C.

Sensitization of patients with diphenylcyclopropenone (DPCP): DPCP was purchased from Professional Compounding Centers of America (C198897, Houston, TX, USA). A single dose of DPCP of 30 μg/cm^2^ (24 μL of 0.125% DPCP in acetone) was applied to a filter paper in a 12 mm Finn Chamber (Item #: AL7014, SmartPractice, Calgary, AB, Canada) and placed on the UVB-exposed skin area immediately after the last UVB exposure (Group 3) or on the non-UVB-exposed skin of the right upper buttock (Group 2). The DPCP patch was removed after 48 h.

Challenge with DPCP: All patients in groups 1, 2, and 3 were challenged with DPCP on the upper inner arm skin. Four weeks after the sensitization (Groups 2 and 3), DPCP was applied to filter-paper-lined, 8 mm Finn Chambers on a 6-chamber patch. Five different concentrations of DPCP in 10 μL acetone were applied: 1.24, 1.98, 3.17, 5.1, and 8.12 μg/cm^2^, plus a control with 10 μL of acetone. The DPCP patches were removed 6 h later.

Measurement of skin thickness: A bi-fold skin thickness was measured using a micrometer (Mitutoyo, Kawasaki, Japan) right before the DPCP challenge (baseline). The second measurement was taken at each of the six DPCP chamber sites 48 h after the challenge. The increase in skin thickness was calculated by subtracting the baseline value. The increase in skin thickness (swelling) is a cutaneous immune response to DPCP (CHS response) and is used as the experimental readout of UVB-induced immunosuppression.

### 2.3. RNA-Seq and Data Analysis

Skin biopsies were stored in RNA later^TM^ Stabilization Solution (Invitrogen, Carlsbad, CA, USA) at −20 °C. RNA was purified using RNeasy^®^ Lipid Tissue Mini Kit (QIAGEN, Germantown, MD, USA) according to the manufacturer’s instructions. RNA samples were sent to Novogene Corporation, Inc. (Sacramento, CA, USA) for bulk RNA sequencing (RNA-seq). After mRNA library construction (poly (A) enrichment), raw reads in FASTQ format were first processed through Novogene’s in-house scripts and then confirmed with our in-house scripts. Homo sapiens (GRCh38/hg38) was used as the reference genome and genome annotation file.

We analyzed differential gene expression (DGE) using raw counts from Novogene’s data in the DESeq2 package in R. No-UVB and UVB samples from the same individuals were compared. Wald statistics were used to evaluate the significance of DGE, with *p*-value adjustment using the Benjamini–Hochberg (B-H) method.

Ingenuity pathway analysis (IPA) was applied for signal network analysis [30]. Canonical pathways were selected based on *p*-value ≤ 0.05 and further supported by activation *z*-score analysis of pathway directional state (≥2 activation; ≤−2 inhibition). To analyze specific pathways, we developed custom molecular networks, such as inflammation and DAMP pathways. Heatmaps were generated using row *z*-scores of the normalized counts, plotting the *z*-score and compacting the edges between −1 and 1. Gene Set Enrichment Analysis (GSEA) was performed using the GSEA software from the Broad Institute, following the standard pipeline [31,32]. We analyzed and replotted (modified “replotGSEA” from Rtoolbox) the running score of the C7 MSigDB gene set “GSE9988_ANTI_TREM1_VS_CTRL_TREATED_MONOCYTES_UP”.

### 2.4. Histochemical and Immunohistochemical Staining of Skin Biopsy Sections

To detect CD163+, p21+, and TREM1+ cells, formalin-fixed paraffin-embedded (FFPE) skin tissues were sectioned (5 μm), and H&E staining was performed by the UAB Comparative Pathology Laboratory. Tissue staining was assessed independently by two people using computer software and manual counting.

The Lunaphore COMET hyperplex workflow was applied to stain paraffin-embedded skin tissue sections (30). It was carried out by the Flow Cytometry Core at UAB. We used anti-CD163 (MRQ-26, Cat: 163M-14, Cell Marque, Rocklin, CA, USA); anti-p21 (12D1, Cat: 2947S, Cell Signaling, Danvers, MA, USA); and anti-TREM1 (Cat: BS10306R, Bioss, Woburn, MA, USA) antibodies. Coralite Plus 647 (α-Rabbit, Cat: RGAR005; α-Mouse, Cat: RGAM005) and Coralite 555 (α-Rabbit, Cat: RGAR003, Proteintech, Rosemont, IL, USA) antibodies were used as secondary antibodies. We subtracted the tissue autofluorescence post-acquisition using HORIZON (v.2.1.0.0) software. One section from each skin sample (7 no-UVB and 7 UVB) was used for multiplexed antibody staining and imaging as described by the manufacturer (Lunaphore, Tolochenaz, Switzerland). The results of CD163, TREM1, and p21 staining were obtained from three different cycles using the same tissue section. The number of positive cells on the section was counted in ImageJ 1.54p with Java1.80_345. Based on intact tissue morphology, two to three fields per section were counted, and the average number of positive cells per field (250 × 150 μm) was calculated from 7 no-UVB or 7 UVB skin sections.

Cyclobutane pyrimidine dimers (CPD) were detected using a previously published protocol with minor modifications [33]. Anti-Thymine Dimer mAb (clone KTM53, Cat: MC-062, Kamiya Biomedical Company, Seattle, WA, USA) and secondary Goat anti-Mouse IgG1 Alexa Fluor™ 488 (Cat: A-21121, Invitrogen, Carlsbad, CA, USA) were used to stain CPD. Sections were mounted with Antifade Mounting Medium (Cat: H-1200, Vectashield, Burlingame, CA, USA). Images were captured using an All-in-one fluorescence BZ-X710 microscope (Keyence, Itasca, IL, USA). The number of positive cells per field was counted in ImageJ, and the average per field was calculated.

### 2.5. Statistical Analysis

We used two-way ANOVA with mixed-effects models and post hoc Tukey tests to evaluate dose-dependent CHS and a one-way ANOVA with post hoc Tukey test for CHS at a specific dose. The two-tailed Fisher’s exact test was applied for CHS in susceptible and resistant individuals. Canonical pathways were analyzed based on *p*-value ≤ 0.05 (Right-Tailed Fisher’s Exact Test) and further supported by *z*-score analysis of pathway directional state (≥2 activation; ≤−2 inhibition). For multiple testing, *p*-values were adjusted using the Benjamini–Hochberg (B-H) method to control false discovery rates. All statistical analyses were performed in GraphPad Prism v10.0; *p*-values ≤ 0.05 were considered significant.

## 3. Results

### 3.1. UVB Induces Immunosuppression, Tissue Damage, and Inflammation in the Skin

Sixty-two of 72 recruited participants completed the study. CHS response (cutaneous immune response) to DPCP was used as the experimental readout of UVB-induced immunosuppression. Group 1 (negative control, 19 patients), which was not sensitized but challenged with DPCP, did not display significant CHS responses (Figure 1A). Group 2 (positive control, 17 patients), which was sensitized and challenged with DPCP, showed significant CHS responses in a dose-dependent manner. In contrast, Group 3 (26 patients), which was irradiated with UVB before sensitization, showed significantly lower CHS responses than Group 2. Histological evaluation of skin biopsies before and after UVB showed that UVB caused necrosis of the upper epidermal layers, neutrophil infiltration below the stratum corneum, apoptotic keratinocytes in the epidermis, spongiosis and mitotic figures, a sparse perivascular and adnexal inflammatory infiltrate, and dermal fibrosis in the skin (Figure 1B).

To examine UVB-induced effects on skin, skin biopsies collected before and 24 h after UVB exposure were subjected to RNA-seq. Initial RNA-seq data analyses showed that UVB significantly modulated 8899 genes (*p* ≤ 0.05) (Figure 1C). Further analysis showed that UVB reduced the gene levels of Langerin (*CD207*) and *IL34* (Figure 1D). In contrast, *IL1β*, *IL10*, *CXCL1*, *TREM1*, *MYD88*, *CD163*, *ARG2*, *PTGS2*, and *CD274* genes were significantly increased by UVB. Furthermore, immunohistochemical staining showed that UVB significantly increased the number of CD163+ and TREM1+ cells in the skin (Figure 1E).

### 3.2. Susceptibility to UVB-Induced Immunosuppression Is Associated with the Level of UVB-Induced Immunosuppressive Inflammation in the Skin

To examine UVB-induced immunosuppression, we compared Group 2 (No-UVB) with Group 3 (UVB-treated). Results showed that seven of 26 (27%) individuals treated with UVB developed CHS after challenge with the highest DPCP dose in Group 3 (Figure 2A), compared with 15 of 17 (88%) individuals showing CHS in Group 2. The two-tailed Fisher’s exact test indicated a significant difference in CHS between Group 3 and Group 2 (*p* = 0.0001).

To link UVB-induced effects in the skin to susceptibility to immunosuppression in Group 3, we collected skin biopsies before and after UVB and subjected the samples to RNA-seq. The IPA showed that UVB significantly modulated 116 specific canonical pathways in susceptible individuals who did not show CHS (UVB-S), whereas 43 specific canonical pathways were significantly modulated in resistant individuals (UVB-R) who showed CHS after the DPCP challenge (an increase in skin thickness above the baseline before the DPCP challenge) (*p* < 0.05). Both groups shared 63 canonical pathways (Figure 2B). UVB modulated more specific canonical pathways in UVB-S than in UVB-R.

The characterization of specific pathways modulated by UVB in UVB-S and UVB-R (*p* ≤ 0.05) showed that UVB modulated a significantly higher level of immune/inflammatory pathways in UVB-S (51/116, 44%) than in UVB-R (7/43, 16%) (Figure 2C). To verify the results, we conducted an unbiased analysis by loading the total RNA-seq data from UVB-S and UVB-R into the GSE9988 C7 immune signature gene set from MSigDB (Molecular Signature Database). Gene Set Enrichment Analysis (GSEA) showed that the hallmark inflammatory response pathway was significantly enriched only in UVB-S, but not in UVB-R (normalized enrichment score: 2.269, *p*-value: 0, FDR: 0, Figure 2D). The results support a higher level of immune/inflammatory responses in UVB-S than in UVB-R. We then analyzed the immune/inflammatory pathways specifically activated in UVB-S (51) or UVB-R (7) (Figure 2C). Results showed that the top 10 significantly modulated signaling pathways with the lowest *p*-values in UVB-S included immunosuppressive pathways such as interleukin-10 signaling and immunoregulatory interactions (Figure 2E). In contrast, none of these pathways were significantly modulated in UVB-R. Moreover, the *z*-scores indicated that cytokine storm, immunoregulatory interactions, and IL-10 signaling pathways were significantly upregulated in UVB-S (*z*-scores > 2, Figure 2F). Interestingly, the seven immune/inflammatory pathways that were significantly modulated in UVB-R did not exhibit significant up- or down-regulation (*z*-scores < 2 or NA, Figure 2F). These results indicate that UVB induces a high level of immunosuppressive inflammation in UVB-S, in contrast to UVB-R.

### 3.3. UVB Modulates DNA Damage/Repair and Cell Cycle/Death Pathways in the Skin at a Similar Level Between UVB-S and UVB-R

To examine whether UVB-induced DNA damage/repair and cell cycle/death were associated with the level of inflammation in the skin and susceptibility to immunosuppression in UVB-S and UVB-R, we detected UVB-induced CPD and p21 (CDKN1A) in the skin. CPD is a marker for UVB-induced DNA damage [14,16], while p21 is a cyclin-dependent kinase inhibitor transcriptionally activated by p53 in response to UVB-induced DNA damage [34,35]. Results showed that normal skin had undetectable CPD+ and a few p21+ cells in the epidermis (Figure 3A,B). UVB induced a high level of CPD+ and p21+ cells in the epidermis, validating UVB-induced DNA damage and effects on cell cycle/death. However, there were no significant differences in either CPD+ or p21+ cells between UVB-S and UVB-R (Figure 3A,B).

In further experiments, we analyzed DNA-related pathways that were significantly modulated in both UVB-S and UVB-R. The analysis showed that UVB significantly modulated 24 DNA damage/repair and cell cycle/death pathways in both UVB-S and UVB-R (Figure 3C). The *z*-score analysis indicated that these pathways were modulated in a similar pattern and at a similar level (Figure 3D). The *z*-score of a pathway represents not only the level but also the directional state (activation or inhibition) of gene expression in the pathway. Among the top 10 pathways, Cell Cycle Checkpoints, homologous recombination repair (HRR) or single-strand annealing (SSA) pathways of homology-directed repair (HDR), and TP53 Regulates Transcription of Cell Cycle Genes are significantly upregulated in an identical pattern in UVB-S and UVB-R (*z*-scores > 2). Collectively, our data did not show a significant difference in UVB-induced DNA damage/repair or cell cycle/death pathways between UVB-S and UVB-R.

### 3.4. UVB-Induced Activation of Damage-Associated Molecular Patterns (DAMPs) Is Linked to UVB-Induced Immunosuppressive Inflammation in the Skin and Immunosuppression

To explore mechanisms underlying differences in immune/inflammatory pathways between UVB-S and UVB-R, we interrogated the pathways that were significantly activated in UVB-S and UVB-R. Results showed that among 51 immune/inflammatory pathways in UVB-S (Figure 2C), nine were related to DAMP/pathogen-associated molecular pattern (PAMP) pathways (Figure 4A). Notably, none of these DAMP/PAMP pathways were significantly modulated in UVB-R. Among the DAMP pathways, the immunological cell death signaling pathway and pyroptosis signaling pathway were significantly up-regulated (*z*-scores > 2, Figure 4B). Moreover, TLR and TREM1 pathways, which are key receptors for DAMPs [36], are significantly up-regulated (*z*-scores > 2, Figure 4B). In further unbiased analysis to verify the results, we loaded total RNA-seq data from both UVB-S and UVB-R onto the C7 gene set “GSE9988_ANTI_TREM1_VS_CTRL_TREATED_MONOCYTES_UP” (MSigDB), which was generated by activating TREM1 signaling pathways with an agonist antibody compared with a control agent. GSEA showed that TREM1 signaling was significantly enriched only in UVB-S but not in UVB-R (Figure 4C). In comparison, we loaded total RNA-seq data from samples before and after UVB onto the same C7 gene set and conducted the same unbiased analysis. Notably, results showed that UVB activated the TREM1 signaling pathway in UVB-exposed samples compared to non-UVB-exposed samples in an equivalent pattern to UVB-S compared to UVB-R (Figure 4D). The results imply that UVB did not significantly activate the DAMP pathway in UVB-R.

## 4. Discussion

The susceptibility to UVB-induced immunosuppression is generally attributed to genetic background [6,9,10,11,37]. Mechanisms remain to be fully elucidated. To address the issue, the current study examines UVB-induced effects in the skin and UVB-induced immunosuppression in the same individuals. We have found that the susceptibility of healthy humans to UVB-induced immunosuppression (UVB-S) is linked to elevated UVB-induced immunosuppressive inflammation and activation of the DAMP pathway in the skin, in contrast to those who are resistant to UVB-induced immunosuppression (UVB-R). The study has added new insights into mechanisms for human susceptibility to UVB-induced immunosuppression and improved our understanding of UVB-induced skin diseases.

Our data show that healthy people have different susceptibilities to UVB-induced immunosuppression (Figure 2A), as in previous reports [6,7,8]. In accordance with the literature [8,28,38], our study shows that UVB induces skin damage, inflammation, and immunosuppression. The analysis of RNA-seq data shows that UVB significantly reduces *CD207*, a marker for Langerhans cells [39] and *IL34*, a growth cytokine for Langerhans cells [40] (Figure 1D). It significantly increases genes of inflammatory cytokines (*IL1b*, *CXCL1*, and *IL10*), myeloid cells (*TREM1*, *MARC1*, and *CD163*), and immunosuppressive molecules (*Arg2*, *PTGS2*, and *CD274*). Immunohistochemical staining supports an increase in TREM1+ and CD163+ myeloid cells in UVB-exposed skin (Figure 1E).

Animal studies demonstrate that the level of UVB-induced inflammation is associated with immunosuppression [7,41]. We have found that the susceptibility to UVB-induced immunosuppression is associated with the level of UVB-induced immunosuppressive inflammation in the skin (Figure 2). Notably, immune/inflammatory pathways are significantly modulated in UVB-S. In contrast, only a few immune/inflammatory pathways are significantly modulated in UVB-R. The unbiased GSEA verifies that the hallmark inflammatory response genes are significantly enriched only in UVB-S, but not in UVB-R. Notably, cytokine storm, immunoregulatory interactions, and IL-10 signaling pathways are among the top five activated immune/inflammatory pathways in UVB-S. These pathways are not significantly modulated in UVB-R. Immunoregulatory molecules PD-1/PD-L1 and cytokine IL-10 have important roles in the regulation of CHS responses [42,43,44,45]. Our study demonstrates that human susceptibility to UVB-induced immunosuppression is linked to the level of UVB-induced immunosuppressive inflammation in the skin.

UVB-induced DNA damage initiates inflammation and immunosuppression in human and animal studies [14,15,16,17,24,46]. A defect in the repair of UVB-induced DNA damage increases the susceptibility to UVB-induced immunosuppression [17,47,48]. In contrast, enhanced repair of UVB-induced DNA damage reduces UVB-induced inflammation in the skin and inhibits immunosuppression [17,25]. Our studies have not detected a significant difference in the number of CPD+ and p21+ cells in the skin between UVB-S and UVB-R. CPD is a marker for UVB-induced DNA damage in skin, and its level is closely associated with UVB-induced inflammation and immunosuppression [14,15,16]. p21 plays important roles in the cell cycle and apoptosis in response to DNA damage [49]. Furthermore, IPA shows that UVB significantly modulates 24 DNA damage- and cell cycle/death-related pathways in both UVB-S and UVR-R, in a similar manner and to a similar extent (Figure 3D). Cell Cycle Checkpoints, homology recombination repair (HRR) or single-strand annealing (SSA) pathways of homology-directed repair (HDR), and TP53-regulated transcription of Cell Cycle Genes, which are important for UVB-induced DNA damage and repair [50], are significantly upregulated in an equivalent pattern in UVB-S and UVB-R (*z*-scores > 2). The results imply that susceptibility to UVB-induced inflammation and immunosuppression does not seem to be associated with sensitivity to UVB-induced DNA damage/repair and cell cycle/death. Additionally, our data suggest that technical issues such as UVB dose variations or undefined patient factors may not be significant.

UVB-induced activation of DAMP pathways plays a role in UVB-induced inflammation [7,26,27]. Our data show that DAMP pathways are significantly activated only in UVB-S, but not in UVB-R (Figure 4). The data show that the Pyroptosis Signaling and Immunogenic Cell Death pathways, which lead to the release of DAMP ligands from stressed or dying cells [7,26,27,51], are upregulated only in UVB-S compared with UVB-R (Figure 4). Moreover, nucleotide-binding oligomerization domain-like receptor (NLR) and inflammasome signaling pathways are also activated only in UVB-S. The activation of these pathways results in pyroptosis, immunogenic cell death, and cytokine storm [52,53]. The literature reports that UVB activates the inflammasome and NLR pathways [54,55,56]. Our data indicate that these pathways are activated only in UVB-S and suggest a mechanism for the high level of inflammation in the skin of UVB-S.

A notable question is why UVB induces a similar level of DNA damage in the skin, whereas the activation of the DAMP pathway is different between UVB-S and UVB-R. DAMP ligands are recognized by pattern-recognition receptors, such as Toll-like receptors (TLRs) and TREM1, thereby triggering the production of pro-inflammatory cytokines [7,26,27]. A defect in the *Tlr4* gene or blocking TLR4 inhibits UVB-induced IL-10, immunosuppression, and skin cancers in animal studies [57,58,59]. TREM1 also serves as a receptor for DAMP ligands and amplifies TLR4-mediated inflammatory responses and cytokine storm [36,60,61]. Our recent studies have demonstrated that blocking TREM1 with an antagonistic peptide inhibits UVB-induced immunosuppression and carcinogenesis [30]. Interestingly, the TREM1 pathway is upregulated only in UVB-S (Figure 4C). In contrast, the TREM1 pathway is not significantly activated in UVB-R. This pattern is consistent with the analysis of UVB-exposed people compared with non-UVB-exposed controls.

Limited information is available for genetic effects on UVB-induced activation of the DAMP pathway in humans. A mouse study shows that TLR3 detects UVB-induced damage and that self-RNA is a damage-associated molecular pattern [62]. There are reports of single-nucleotide polymorphisms (SNPs) in the TLR4 gene, such as Asp299Gly, which alter TLR4 function in response to its ligands and are associated with various infectious and non-infectious diseases [63,64,65,66], and with a decreased incidence of skin cancers [67,68]. Reports also suggest that the *TREM1* gene SNPs are associated with various diseases [69,70]. However, it is unknown whether *TLR4* or *TREM1* SNPs affect UVB-induced inflammation or immunosuppression. Although there are reports of other SNP effects in human skin cancers [71,72,73], limited evidence is associated with UVB-induced effects. Further studies are required to examine specific genetic factors responsible for susceptibility to UVB-induced immunosuppression in humans and determine the molecular mechanisms for UVB-induced activation of DAMP pathways in UVB-S and UVB-R.

There are some limitations to our study. Despite statistically significant differences in CHS and signaling pathways between UVB-S and UVB-R, the current study had a limited number of skin biopsies due to the small number of UVB-R individuals (seven out of 26, 27%). An increase in the number may provide genetic evidence for differences in the activation of DAMP pathways between UVB-S and UVB-R and add more signaling pathways or molecules to the mechanistic analysis. Like most previous reports, our study collected one biopsy before and one after UVB radiation. There is an IRB concern about the increased risk to participants from UVB exposure on multiple sites and multiple biopsies.

## 5. Conclusions

UVB-induced activation of DAMP pathways is linked to UVB-induced immunosuppressive inflammation in the skin and susceptibility to UVB-induced immunosuppression in humans (Scheme 2). Although animal studies have demonstrated that the pattern recognition receptors TLR4 and TREM1 in dendritic cells are required for UVB-induced immunosuppression, future single-cell-level studies will validate mechanisms underlying UVB-induced activation of DAMP pathways in specific human cells.

## Figures and Tables

**Scheme 1:**
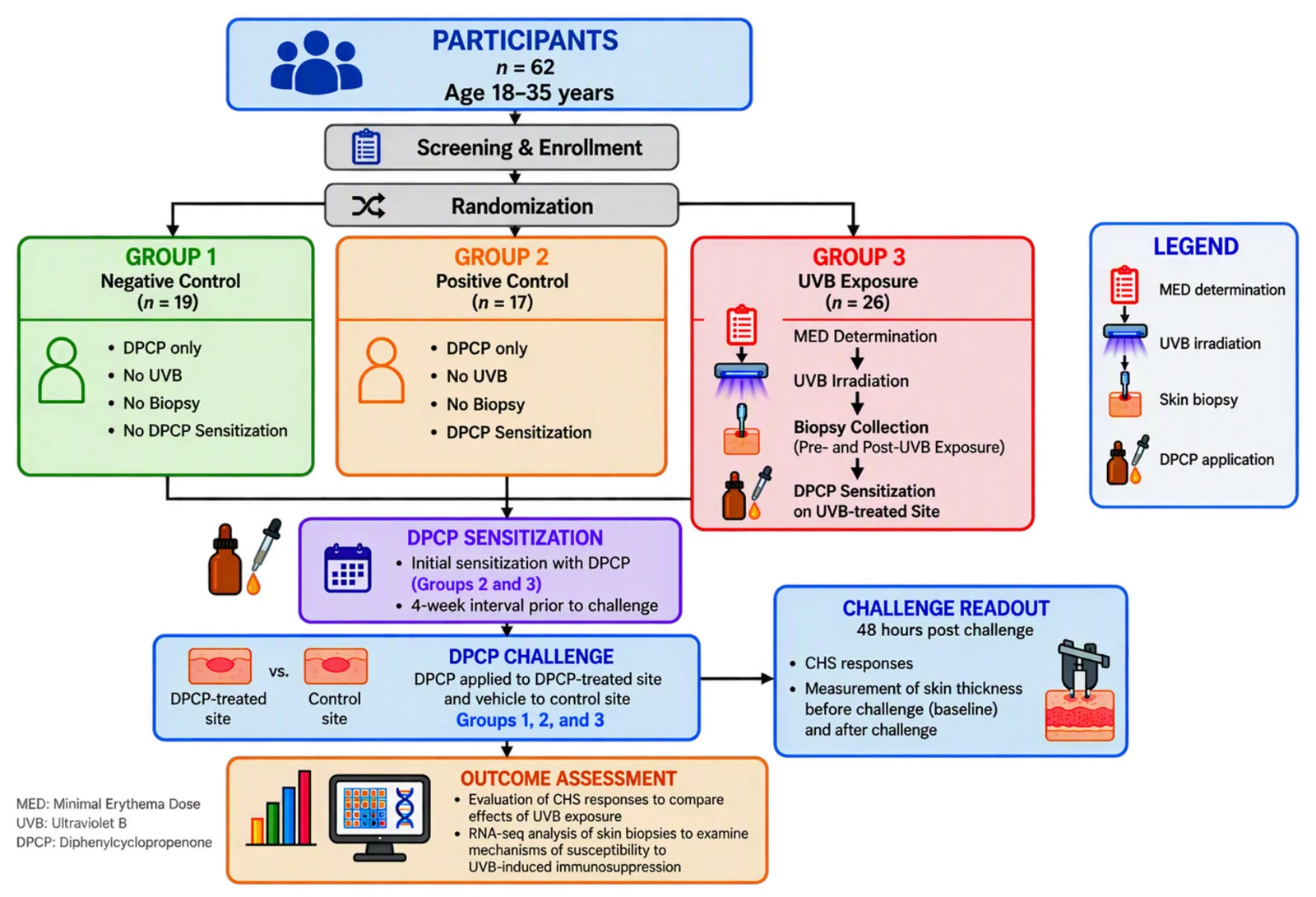
Overview of the participants, study design, methods, and outcomes.

**Figure 1:**
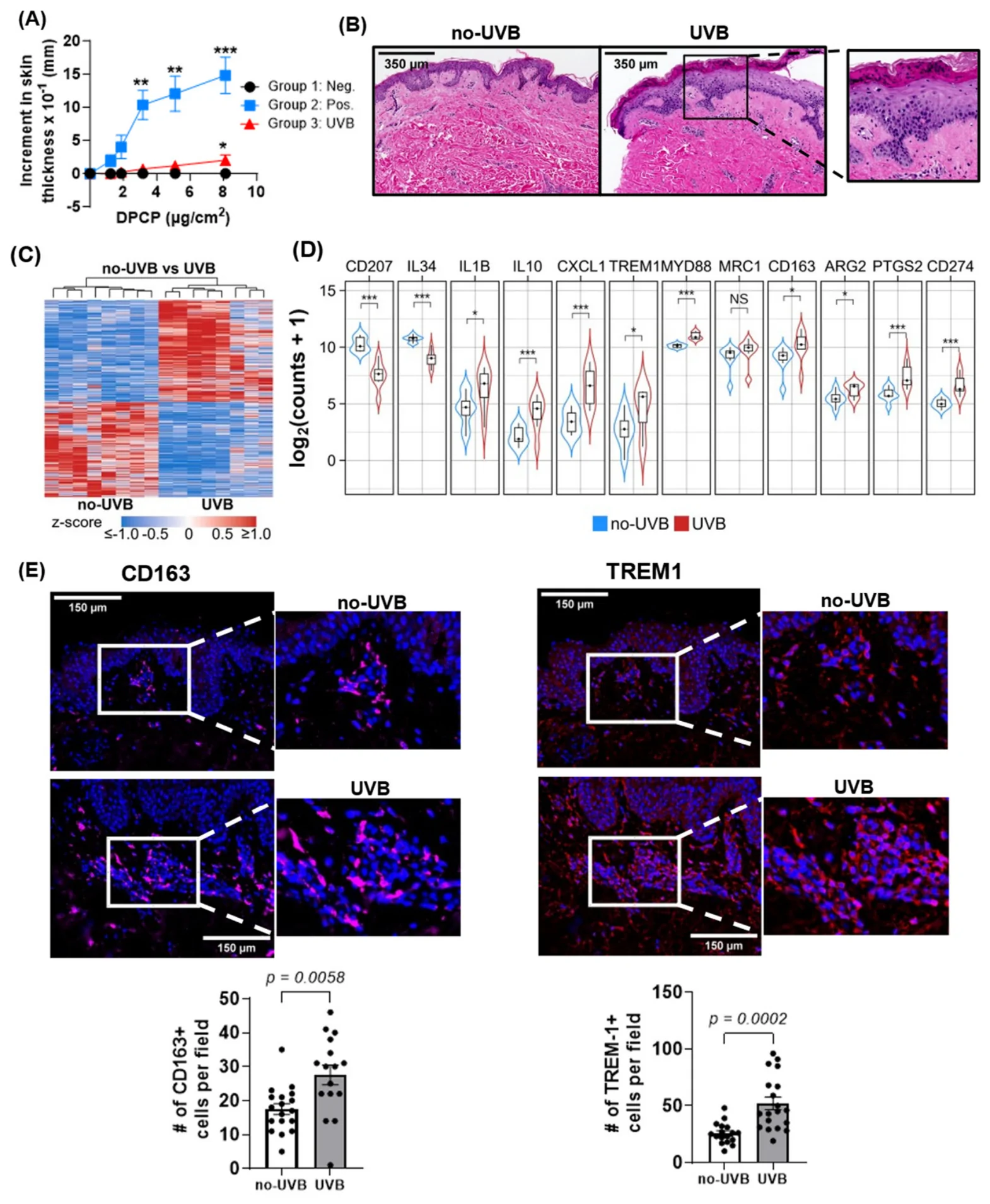
**UVB-induced immunosuppression is associated with UVB-induced inflammation and increased myeloid cells in the skin.** (**A**) CHS responses are inhibited by UVB exposure. We measured skin thickness following the challenge with various doses of allergen (DPCP) as described in Table 1. Mixed-effects analysis with post hoc Tukey’s test for significance *** *p* ≤ 0.001, ** *p* ≤ 0.01, * *p* ≤ 0.05. (**B**) UVB induces skin damage. The representative images show the phototoxic effects on human skin tissues. The zoomed-in area shows epidermal damage induced by UVB. (**C**) The heat map of RNA-seq data shows the significant modulation of genes in skin tissues after UVB compared to before (no-UVB) (*padj* ≤ 0.05) (*n* = 16, 8 no-UVB, 8 UVB). (**D**) UVB significantly reduces Langerhans cell marker *CD207* (Langerin) and growth factor *CD34*, whereas it significantly upregulates gene expression of inflammatory (*IL1b*, *CXCL1*, *Mac1*, *CD163*, *TREM1*, and *MyD88*) and immunosuppressive (*IL-10*, *Arg2*, *PTGS2*, and *CD274*) factors. The Wald test was used to evaluate differential gene expression. *** *p* ≤ 0.001, * *p* ≤ 0.05. (**E**) Immunohistochemical staining shows UVB-induced increases in CD163+ and TREM1+ cells in the skin. Lunaphore COMET^TM^ hyperplex workflow was applied to detect positive cells. Representative images from the same field of skin samples show co-localization of CD163+ and TREM1+ cells before and after UVB. Based on intact tissue morphology, two to three fields per section were counted, and the average number of positive cells per field (250 × 150 μm) from 7 no-UVB or 7 UVB skin samples was calculated. Welch’s two-tailed unpaired *t*-test was used for statistical analysis.

**Figure 2:**
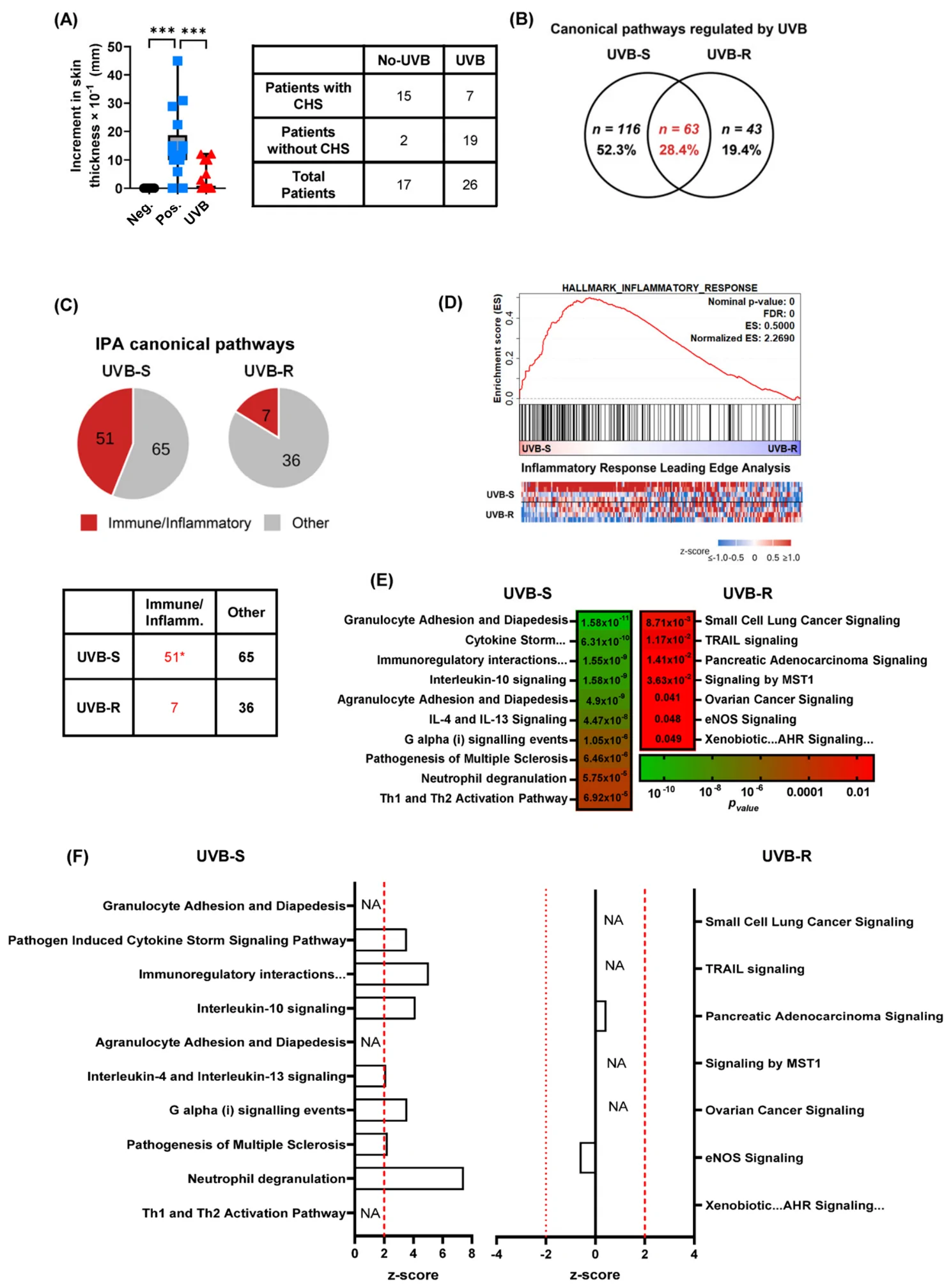
**UVB-induced immunosuppression is associated with a high level of UVB-induced immunosuppressive inflammation in the skin.** (**A**) UVB induces immunosuppression in a portion of participants. At the highest dose of DPCP for the challenge, 7 out of 26 in the UVB-exposed group (UVB) developed CHS, compared to 15 out of 17 in the positive group (no-UVB). Mixed-effects analysis with post hoc Tukey’s test shows statistical significance, *** *p* ≤ 0.001. Two-tailed Fisher’s exact test for the contingency table yields a statistical *p*-value of 0.0001 between no-UVB and UVB. (**B**) Skin biopsies from individuals susceptible (UVB-S) or resistant (UVB-R) to UVB-induced immunosuppression (Group 3) were collected before and after UVB radiation and subjected to RNAseq analysis. IPA analysis reveals that UVB-S and UVB-R patients share 63 pathways but also exhibit specific pathways, 116 and 43, respectively (*p* ≤ 0.05)(*n* = 4 for each). (**C**) The pie chart shows that after UVB radiation, 51 immune/inflammatory pathways in UVB-S and 7 in UVB-R are significantly modulated (*p* ≤ 0.05). Two-tailed Fisher’s exact test indicates a significant difference between the two groups (* *p* = 0.0014). (**D**) GSEA analysis shows that after UVB radiation, the inflammatory response hallmark is significantly enriched only in UVB-S, but not in UVB-R (FDR: <0.001). (**E**) The top 10 modulated signaling pathways with the lowest *p*-values out of 51 in UVB-S and 7 in UVB-R are listed based on *p*-values (*p* ≤ 0.05). (**F**) *Z*-scores of the top 10 signaling pathways. The red dotted lines indicate the activation threshold (*z*-score = 2). NA: The *z*-scores are not available due to insufficient evidence to predict the direction of pathway modulation.

**Figure 3:**
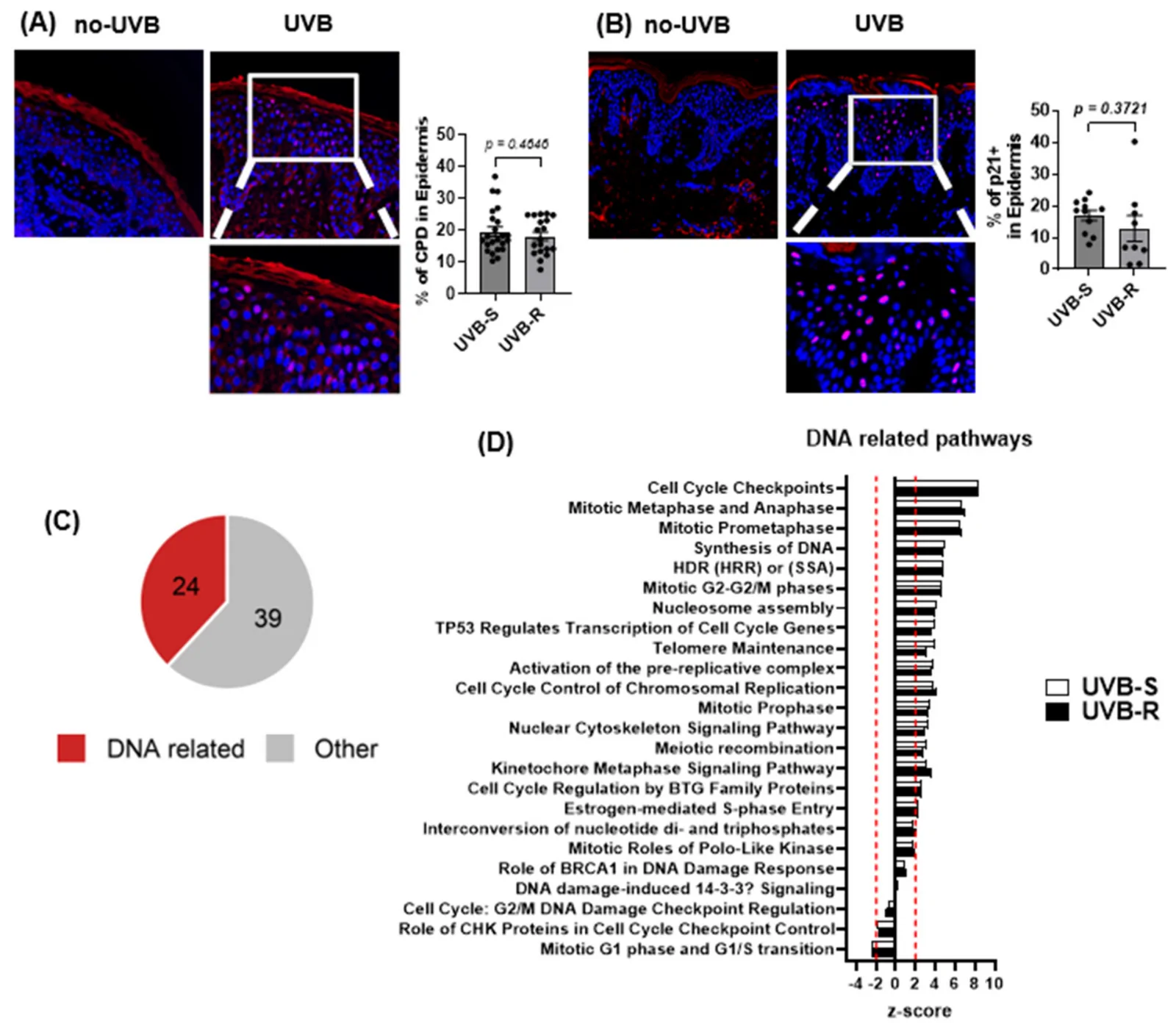
**UVB induces similar levels of DNA damage/repair and cell-cycle/death pathways in the skin of UVB-susceptible (UVB-S) and -resistant (UVB-R) individuals.** Skin biopsies were collected 24 h after UVB for immunohistochemical staining. Results show a similar level of CPD+ (**A**) or p21+ (**B**) cells in the epidermis. The positive cells were counted microscopically, and the percentage of positive cells per field was calculated based on the total number of DAPI+ (nucleus) cells in the epidermis (*n* = 9−23 fields). Welch’s unpaired *t*-test was applied for statistical analysis. (**C**) The pie chart shows that among the 63 significantly modulated pathways shared by UVB-S and UVB-R, 24 are DNA damage/repair and cell cycle/death-related pathways. (**D**) The bar graph shows similar *z*-scores of the 24 pathways between UVB-S and UVB-R. The red line represents the inhibition or activation threshold (*z*-score ≤ −2 or ≥2).

**Figure 4:**
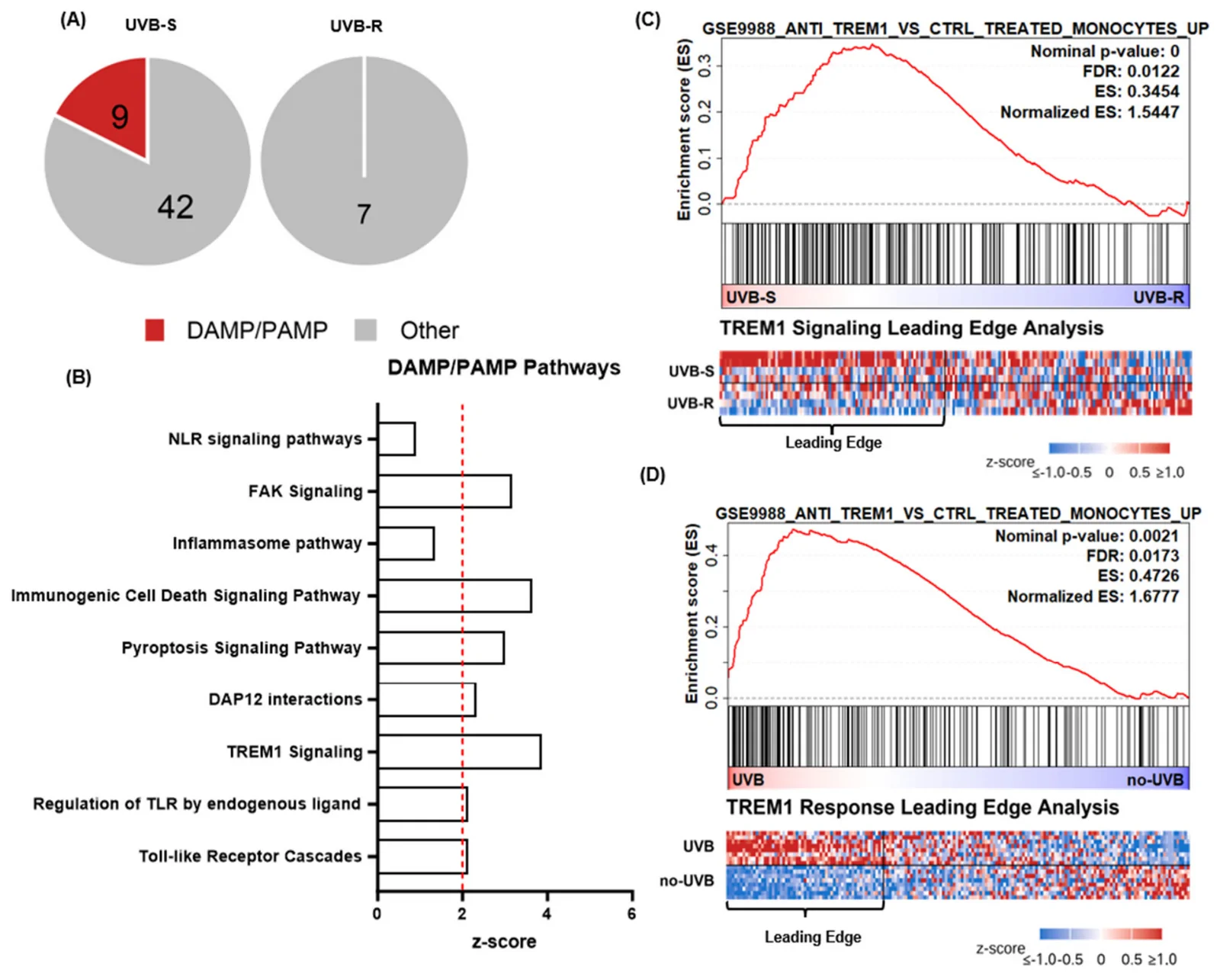
**UVB-induced activation of the DAMP pathway is linked to UVB-induced immunosuppression.** RNA-seq analysis of skin biopsies is described in Figure 2. (**A**) A pie diagram shows that among the 51 significantly modulated pathways in UVB-susceptible (UVB-S) patients, 9 DAMP/PAMP-related pathways are significantly activated. In contrast, among the 7 significantly modulated pathways in UVB-resistant (UVB-R) patients, none of the DAMP/PAMP pathways are activated. (**B**) *Z*-scores of the DAMP/PAMP pathways in UVB-S. (**C**) GSEA analysis shows that the TREM1 signaling pathway is significantly enriched only in UVB-S compared to UVB-R based on the data set of TREM1 activation with a TREM1 agonist compared to a control agent. (**D**) GSEA analysis shows that the TREM1 signaling pathway is significantly enriched in UVB-treated skin samples compared to no-UVB-treated controls based on the data set of TREM1 activation with a TREM1 agonist compared to a control agent.

**Scheme 2:**
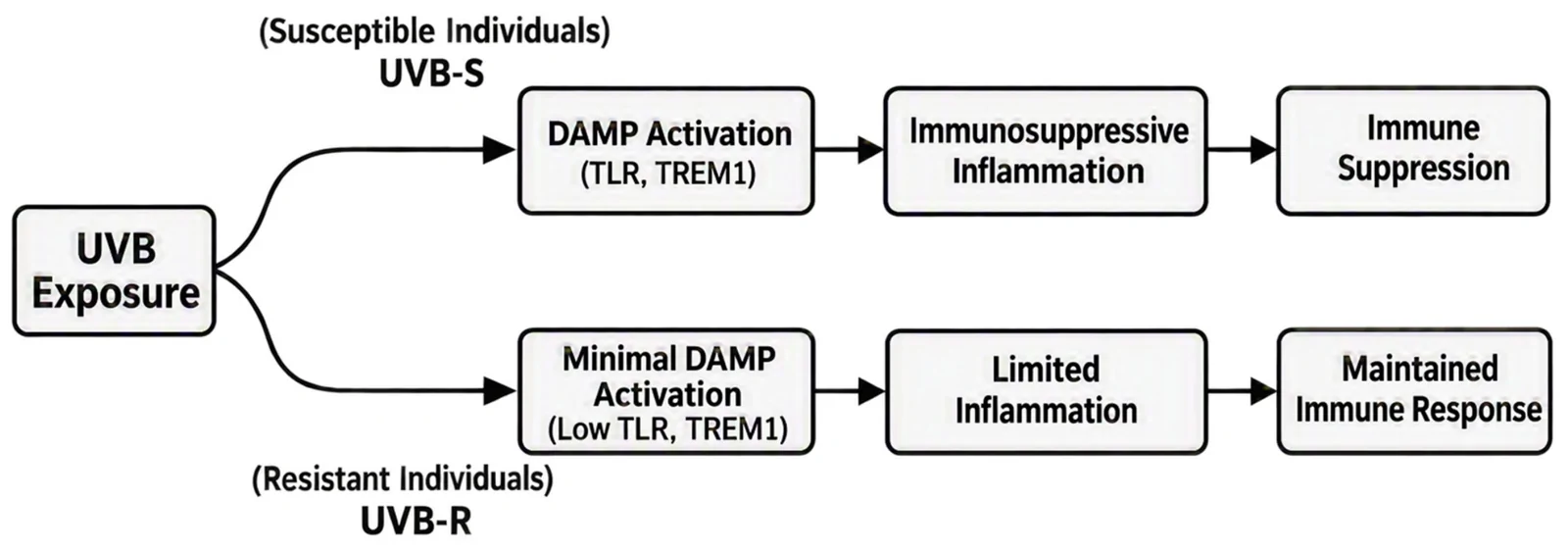
UVB exposure induces divergent responses in susceptible and resistant individuals. In susceptible individuals, UVB enhances damage-associated molecular pattern (DAMP) signaling, including TLRs and TREM1, promoting immunosuppressive inflammation and downstream immune suppression. In resistant individuals, DAMP activation remains minimal, resulting in limited inflammation and preservation of immune responses.

**Table 1:** Participants and study design.

Group ^1^	Age	Males/Females	MED ^2^	UVB ^3^	Biopsy ^4^	Sensitization ^5^	Challenge ^6^
1	20–34 (25.7 ± 3.9)	7/12	No	No	No	No	Yes
2	19–35 (25.8 ± 4.2)	5/12	No	No	No	Yes	Yes
3	19–32 (25.3 ± 3.4)	10/16	Yes	Yes	Yes	Yes	Yes

^1^. Participants were screened and assigned randomly into three groups: Group 1 (Negative Control), Group 2 (Positive Control), and Group 3 (UVB-exposed). The race of all participants was white. ^2^. Six, one-inch area squares of the upper left buttock were exposed to 5 gradually increasing doses of UVB, and one area was left untreated as a non-UVB-exposed control. Skin redness was read with a colorimeter 24 h later, and MED was determined as described. ^3^. Based on MED, two marked sites on the upper right buttock were exposed to 2 MED of UVB for 4 consecutive days. ^4^. Twenty-four hours after the last UVB exposure, two 6mm punch skin biopsies were taken, one from the UVB-exposed skin and the other from the non-UVB-exposed skin area. ^5^. A single dose of diphenylcyclopropenone (DPCP) of 30 μg/cm^2^ (24 μL of 0.125% DPCP in acetone) was applied to a filter paper in a 12-mm Finn Chamber and placed on the upper buttock (Group 2) or on the UVB-exposed skin immediately after the last UVB (Group 3). It was removed after 48 h. ^6^. Four weeks after the DPCP sensitization (Groups 2 and 3), the baseline of bi-fold skin thickness of the upper inner arm was measured using a micrometer. Group 1 without DPCP sensitization was included as a negative control. All groups were challenged with five different doses of DPCP on a 6-chamber patch, along with acetone as a control. The DPCP patch was removed after 6 h. The second measurement of bi-fold skin thickness was performed 48 h after the DPCP challenge at the 5 DPCP-applied and acetone-only sites.

## Data Availability

We are in the process of obtaining permission from the university IRB and will deposit the data after approval. The data underlying this article will be shared on request to the corresponding authors.
